# Exploring nurse practitioners’ collaboration with general practitioners in Norwegian homecare services: a qualitative study

**DOI:** 10.1080/02813432.2024.2381064

**Published:** 2024-07-22

**Authors:** Lene Apeness Kjær, Mette Tøien, Linn Hege Førsund

**Affiliations:** a University of South-Eastern Norway, Drammen, Norway; b Western Norway University of Applied Sciences, Førde, Norway

**Keywords:** Nurse practitioner, general practitioner, collaboration, trust, homecare services

## Abstract

**Objective:**

Nurse practitioners (NPs) have recently been introduced in Norwegian homecare services. The NP role is still in an early implementation phase without standardized role descriptions. NPs are dependent on collaborating with general practitioners (GPs) in the care and treatment of patients. However, little is known about how NPs in Norway experience this collaboration. This study aims to explore how NPs working in homecare services describe their collaborative experiences with GPs, and what influence this collaboration.

**Design:**

The study had a qualitative descriptive design, applying individual, semi structured interviews to generate data from five Norwegian nurse practitioners working in homecare services. Data were analyzed using systematic text condensation.

**Findings:**

The NPs had varied experiences regarding the collaboration with GPs. NPs stated their role as unclear, lacking standards and job descriptions. The NPs experienced that some GPs were uncertain about the NPs competence, which inhibited collaboration and restricted the NPs utilization of their full capability.

NPs experienced a higher degree of collaboration with GPs they knew, and they indicated that trust was the key to facilitate collaboration. The NPs also noted the challenges of establishing relationships with GPs due to the lack of formal meetings and the physical separation of their workplaces.

**Conclusion:**

Interpersonal dynamics, organizational structures and systemic frameworks influenced the collaboration between GPs and NPs in homecare services. Trust was identified as an important prerequisite for collaboration.

## Introduction

The primary healthcare system in Norway is facing significant challenges due to the increasing number of patients with advanced and complex health issues. This is particularly evident in homecare where advanced and comprehensive nursing skills are crucial [1,2]. To address this, the role of ‘nurse practitioner’ (NP) was introduced in primary healthcare services as part of measures outlined in the White Paper Meld. St. 26 – ‘The future primary healthcare service – proximity and entirety’ (2014–2015). The White Paper addresses proposals for evolving the municipal health and care services to effectively meet present and future challenges. NPs undergo master’s degree education to develop advanced nursing skills, clinical assessment expertise, decision-making abilities, and professional competence. This educational background enables them to assume independent roles within primary healthcare services [3]. The focus in this study is on the NPs in homecare.

The organizational structure of the Norwegian health care system is built on the principle of equal access to services, independent of social status, location and income. The municipalities are responsible for the primary health care system including nursing homes and homecare [4]. Residents have the right to choose their own GP and are encouraged to see their chosen GP for most health concerns. GPs in Norway act as gatekeepers to other parts of the healthcare system. They coordinate referrals to specialists and hospitals when necessary [4]. Norwegian GPs typically operate in private practices, while some are employed by the municipality. Nevertheless, the majority of the funding for their GP practice comes from the government. In addition, the patients pay a nominal fee for each visit to their GP. Furthermore, the government sets regulations and fee schedules. In Norway the fee schedules are directly linked to GP as a profession. This means that the GP must see the patient to trigger a consultation fee and thus maintain their income base [4].

It’s important to note that NPs in Norway do not have the authority to prescribe medications, refer patients to specialists, or make medical decisions on their own. GPs are responsible for the medical aspects of patient care. To provide comprehensive treatment and care, NPs depend on effective collaboration with general practitioners to ensure holistic patient care [5].

Establishing professional collaboration is vital for effective cooperation [6]. Nevertheless, research suggests that achieving such collaboration can be challenging and sometimes inadequate [5,7]. The healthcare system is a complex collaborative environment influenced by various factors [8]. International studies have indicated that unclear definitions of the NP role can hinder understanding of their scope of practice and create ambiguity regarding legal and medical responsibilities, which can impede collaboration. Conversely, research has found that a positive, respectful, and trusting relationship between NPs and GPs, along with effective communication and interpersonal compatibility, can facilitate cooperation [9,10].

In Norway, where the implementation of the NP role is still in its early stages and lacks established models, there is limited research on collaboration between NPs and GPs. This study aims to explore NPs collaborative experiences with GPs in homecare in Norway. Such knowledge is critical for the future implementation of the NP role in the best interests of patients. The research question guiding this study is: ‘How do NPs describe their collaborative experiences with GPs, and what influence this collaboration?’

## Materials and methods

### Study design

A qualitative design is well-suited for the exploration of personal experiences within less-explored subjects [11]. In line with the aim of our study, we employed a qualitative descriptive design, utilizing semi-structured interviews with NPs to gain insights into their collaborative experiences with GPs. Our data analysis followed a systematic text condensation approach described by Malterud [12].

### The authors’ backgrounds and prior understanding

The first author, LAK, is an experienced NP with a background in collaborating with GPs within homecare. The second author (MT) and the last author (LHF) are registered nurses (RNs) and associate professors with experience of professional practice in homecare, complemented by their research activities in this field. This collective expertise underpinned our research efforts. LAK assumed the principal role in data collection and analysis, while the co-authors actively participated in and provided valuable insights throughout all stages of the research process. Their contributions were also significant in developing both the study design and the manuscript.

### Sample

We employed a purposeful sampling approach to ensure that our informants possessed a deep understanding of the specific subject area, aligning with the methodology outlined by Malterud [12]. Our inclusion criteria pursued NPs in Norway with a master’s degrees who worked with patients in homecare.

For participant recruitment, we adopted a self-selection method, extending invitations through a dedicated Facebook group for NPs with a total of 133 members, including NP students. Although eight individuals initially expressed interest, three were subsequently excluded for not meeting the specified inclusion criteria. Consequently, our final sample comprised five female NPs, each working in different urban municipalities in South-Eastern Norway, with professional experience ranging from 1 to 5 years as NPs.

### Data collection

We employed a data collection method involving five individual semi-structured interviews, a method chosen to facilitate the acquisition of in-depth and comprehensive insights into each participant’s experiences, in line with the approach recommended by Malterud [12]. The interview guide consisted of seven open-ended questions, rigorously tested and refined through a pilot interview. While all participants were asked the same introductory questions, the sequence of subsequent inquiries varied to ensure a dynamic conversation. Informants were actively encouraged to provide specific examples of their collaborative interactions with GPs, and follow-up questions were introduced as needed. This approach allowed informants to describe their unique experiences, resulting in rich and detailed accounts, aligning with Malterud’s methodology.

The interviews were conducted by the first author, LAK. The audio-recorded interviews lasted between 50 and 80 min and were transcribed verbatim. The informants were given the flexibility to choose the interview location, whether it be their workplace or another designated venue. 3 interviews were conducted at the respective informants’ workplaces, 2 were conducted at the University of South-Eastern Norway.

### Analysis

The analysis followed Malterud’s systematic text condensation (STC) method [11], ensuring a structured and rigorous approach to data interpretation. The process unfolded in four distinct steps; (1) total impression – from chaos to themes; (2) identifying and sorting meaning units – from themes to codes; (3) condensation – from code to meaning; and (4) synthesizing – from condensation to descriptions and concepts.

The analysis was initiated by all authors through reading the interviews to familiarize with the material and to obtain an overall impression. We identified preliminary themes representing our first impression of the material and discussed until consensus about six preliminary themes: Recognition and acceptance, legal rights, time and accessibility, face-to-face time, communication, and trust and respect. LAK then proceeded with the analysis, collaborating with MT and LHF throughout each step. LAK first examined the interviews line by line to identify meaning units that described the preliminary themes in various ways. The meaning units were subsequently systematized by naming them with codes. The next step of the analysis entailed condensation of meaning units representing the same codes, before the coded condensates were grouped, synthesized, and categorized [11]. Table 1 provides examples of the steps of the analysis.

**Table 1.** Examples of the steps of the analysis.

Meaning unit	Condensation	Code	Category
‘Because that’s often what they want…. .they want you to be able to justify your findings or the assessments you’ve made. As long as you do that, you demonstrate your competence, you show that they can have confidence in your professional level’ (id 1)	GPs want reasons for findings and assessments	Importance of demonstrating clinical competence in order to promote trust.	Interpersonal dynamics
‘I think it’s the time pressure that’s the greatest problem. That’s my view because they are swamped with work. They were given so many more tasks after the Coordination Reform and their patient lists are far too long’ (id 3)	Believe GPs want cooperation but are prevented by the volume of work tasks	Importance of having time for cooperation	Organizational structures
‘But the role of NP has been expanded to include making more independent assessments, but at the same time I find that I’m bound hand and foot in that we have no prescription rights and no right of referral ‘(id 2)	No prescription rights and no right of referral	Importance of prescription rights and requisition rights to allow NPs to develop their full potential	Systemic frameworks

## Ethics

The study strictly adhered to the principles of the Helsinki Declaration [13]. Prior to the interviews, informants received oral and written information about the aim of the research, the voluntariness of participation and their rights to withdraw. Informed consent was obtained from all participants. The handling and protection of data received approval from the Norwegian Agency for Shared Services in Education and Research (Sikt), with reference number 790053. Confidentiality and anonymity of participants were rigorously upheld throughout the study.

For the reporting of our study, we followed the guidelines outlined in the Consolidated Criteria for Reporting Qualitative Studies (COREQ) [14].

## Results

The results of the analysis provide a comprehensive description of NPs collaborative experiences with GPs in community care and how these are influenced by three key dimensions: interpersonal dynamics, organizational structures, and systemic frameworks.

**Figure 1.**
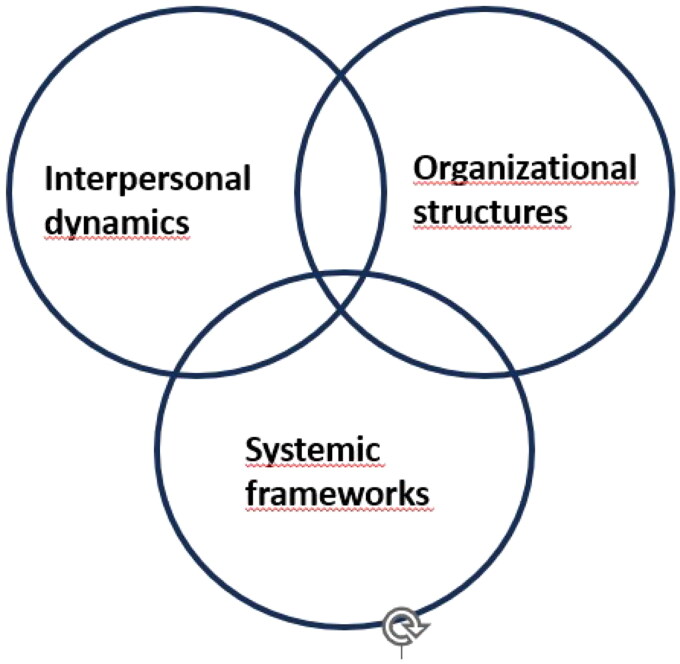
The interwoven key dimensions influencing collaboration between NPs and GPs.

Interpersonal dynamics involve the interactions and relationship between NPs and GPs. Organizational structures encompass aspects such as resources in form of time, places and procedures for collaboration, and systemic frameworks include legal framework for professional practice. The three dimensions are interrelated dimensions (see Figure 1). It is the relationship between them that affects collaboration. Trust is found to be a central theme that permeates collaboration in all three dimensions.

### Interpersonal dynamics

Establishing a solid relationship, both on a personal and professional level, emerged as a crucial factor in fostering trust between NPs and GPs, significantly impacting their collaborative dynamics. Our informants emphasized that the quality of collaboration with GPs varied based on this fundamental element. NPs sought acknowledgment and respect for their expertise and potential contributions when working alongside GPs in patient care. Informants noted that they often received such recognition when conducting procedures like blood pressure measurements or wound management in patients’ home.

However, NPs also revealed instances of trust issues stemming from certain GPs, particularly concerning their assessments, observations, and professional clinical judgments. Given their ability to assess patients in their home environment on a daily basis, NPs possessed valuable insights into patients’ normal conditions and could readily detect signs of change. Regrettably, these valuable insights often went unused, as many GPs rarely sought this information and instead preferred to rely on their own assessments.

Informants believed that one of the primary reasons for this lack of trust in NP assessments was the GPs limited knowledge about their professional competence. This notion was based on the experiences of informants who had established relationships with specific GPs. In such cases, these GPs were more acquainted with the NPs roles and capabilities, consequently demonstrating greater confidence in their observations and assessments compared to GPs they had less familiarity with. Informants perceived this familiarity as a significant influencer on collaboration, as it was noticeably easier to collaborate with those with whom they had a personal relationship. As one of our informants succinctly put it,

‘We collaborate with …. I think about 46 GPs. So it varies an awful lot….some we know better than others, so some really trust us and will also contact us to ask for assessments directly, while others are very sceptical to our role and prefer to carry out assessments on their own. And this is quite understandable when you have the medical responsibility’ (id 2).

The informants articulated a desire for a stronger level of trust in the competence of NPs from GPs and compared this with more established roles in the healthcare system. One informant articulated this attitude, emphasizing,

‘The fact that GPs trust dementia coordinators and physiotherapists, for example, and delegate assessments and observations to them, demonstrates their confidence in their training’ (id 5).

Informants believed that if GPs had more opportunities to become familiar with NPs, it would facilitate a deeper understanding of the potential of the NP role, ultimately making them more open to collaborative efforts. However, they acknowledged that building this familiarity could be a gradual process. Notably, informants pointed out the challenge of limited interaction between GPs and NPs due to their separate work locations, which extended the time required for mutual understanding to develop. One of the informants remarked,

‘I’ve always believed that our lack of face-to-face encounters significantly prolongs the time it takes for them to become familiarized with us’ (id 4).

In the informants’ view, establishing a shared meeting place would be instrumental in helping GPs to better understand NPs and build trust in their professional capabilities. As expressed by an informant:

‘I have had lunch together with the doctors and explained a bit about how we work, and then they’ve understood that we’re experienced RNs that they can trust and so on. Then once you’ve met them, it’s fine, and they know who you are. And actually, they may call you on the phone and say: Can you pay a visit now? I’m very busy today, but she has had a fall. Could you go out there and check if I need to pay her a visit, and then you can call me afterwards?’ (id 4).

### Organizational structures

The analysis revealed that trust and collaboration between NPs and GPs depended not only on interpersonal dynamics but also on organizational structures. NPs served patients within specific regions, while GPs often had responsibilities covering larger geographical areas. Consequently, NPs frequently engaged with multiple GPs within their regions. NPs and GPs were also separated organizationally in different departments. Several informants underscored a contrast between this organizational structure and the more integrated structures seen in hospitals and nursing homes where GPs and NPs/nurses shared the same departments. In these settings, collaboration practises were better established. Informants attributed the variations in trust levels between GPs and NPs in community nursing services versus hospitals or nursing homes to this distinct organizational frame. Doctors in hospitals tended to rely more on the observations of registered nurses (RNs), they said, which seemed to cultivate greater trust. One informant articulated this:

‘In hospitals, doctors place more trust in RNs because they are familiar with them and comprehend their capabilities’ (id 5).

The organizational separation between GPs and NPs in the municipality presented more challenges for informants. They found it difficult and time-consuming to reach out to GPs, and desired more open communication and faster responses, which they considered vital for effective collaboration. Informants primarily communicated digitally with GPs, but many GPs signalled they could not expect rapid responses due to their heavy workloads. As one informant explained:

‘It’s not just a matter of making a phone call to ask about things as you might do if you worked somewhere where there are doctor’s visits’ (id 4).

Consequently, despite the willingness of GPs to collaborate, the absence of shared meeting spaces, established communication channels, infrequent interactions, and time constraints hindered genuine collaboration opportunities, leading some informants to question whether GPs actually had time to collaborate effectively. Informants suggested that an alternative organizational approach, such as permanently assigning a GP to the homecare, could enhance collaboration conditions. They also emphasized the importance of creating shared meeting spaces to facilitate discussions on professional matters and challenges, potentially promoting greater trust.

### Systemic frameworks

The findings revealed that systemic frameworks also played a significant role in influencing collaboration opportunities between NPs and GPs. According to the informants, the systemic factors influencing collaboration were mainly associated with the legal framework for professional practice.

According to the informants, systemic factors included a lack of clarity regarding the NPs role and responsibilities, particularly in terms of how tasks could be shared between doctors and NPs. While some GPs were open to collaboration, the informants noted that others were cautious about maintaining ‘professional boundaries.’ As expressed by one informant,

‘Certainly, they can see that we can be a resource in the follow-up of patients in collaboration with them’ (id 1).

Furthermore, the informants raised concerns about the implementation of legal rights, such as prescription and referral rights, not keeping pace with the evolving NP role. They believed this hindered collaboration. One informant provided an example:

‘As the GP has to see the patient in their office anyway, in order to write a referral, they say that the NP really just represents an extra step’ (id 1).

To enhance patient flow, task allocation, and the utilization of NP expertise, the informants suggested potential measures, including limited prescription and referral rights. According to the NPs, granting the ability to admit patients to a municipal acute unit or hospital in specific cases and referring patients for selected diagnostic imaging tests could foster improved collaboration and task allocation. The informants believed that increasing GPs understanding of how NPs could contribute and what role they could play would lead to greater trust and enabling collaborative task allocation within the NPs area of expertise. Trust was considered pivotal for collaboration.

## Discussion

The aim of this study was to explore NPs’ collaborative experiences with GPs in homecare in Norway. The results showed how interpersonal dynamics, organizational structures, and systemic frameworks collectively influence the trust and collaboration dynamics between NPs and GPs in homecare.

## The importance of knowing one another

The NPs in our study emphasized the importance of the GPs trust as a prerequisite for good collaboration. The informants found that it was easier to collaborate with the GPs they knew. These findings are consistent with international studies which show that GPs who have worked with NPs earlier have a more positive attitude to collaboration with them [15]. Studies of trust in community health care found that a spontaneous trust in health care personnel was related to role and competence, while a deeper trust depended on interpersonal attributes [16,17]. These findings are consistent with the content of the concepts ‘competence trust’ and ‘companion trust’, which states that companion trust can only be earned through experiences within a relation over time [18].

The lack of meeting places made it difficult for NPs to get to know the GPs. International studies attribute problems in collaboration to precisely this lack of meeting places for professional discourse and knowledge exchange – such meeting places can promote a shared understanding among professional practitioners [15]. In Norway results from research exploring how GPs and oncology nurses (ONs) experience their collaboration revealed that the ideal cooperation was ‘meeting of experts’ for discussions and support of each other [19]. Talking together is perceived as the optimal form of collaboration. Not knowing each other, lack of meetings and e-messages in replacement of ad hoc conversations and scheduled meetings obstructed optimal collaboration. Electronic communication can be convenient and suitable for minor queries, but cannot replace talking together [20]. Face-to-face relationships are described as important for successful interaction, even though it is arguable whether this is a realistic option in all situations in the health service [5,21]. The organization of the GP scheme and the community nursing service as separate bodies in the primary health service means that both the organizational and geographic divides are considerable and not well-adapted to personal meetings. A heavy workload also limits opportunities to allocate time to such meetings, in line with the findings of the review of Schadewaldt et al. [22] that compared international research on the experiences of NPs and GPs in relation to collaboration.

Co-location can be highly beneficial to collaboration. This is also recommended in a Norwegian white paper on primary care [1,20]. NPs could be assigned to GP services, forming a team to serve the GPs patient list and dividing tasks based on patient needs. This model is currently being tested in a Norwegian municipality, where NPs collaborate with municipally employed GPs in managing patient lists. While not yet scientifically evaluated, initial user surveys indicate patient and collaborator satisfaction. Currently, another collaborative model is being tested in Norway, but it does not include NPs [23]. An Australian study investigating a new collaborative practice model [22] found that despite structural and policy challenges, personal commitment and the capacity to develop efficient collaboration models were key to ensuring successful NP-GP collaboration. Crucially, the study concluded that face-to-face meetings between NPs and GPs were essential for this success. By fostering physical interactions and professional discussions, NPs and GPs can create familiarity, leading to increased trust, improved collaboration, and ultimately, enhanced patient care.

## The importance of knowledge of the NP’s competence and role

In addition to the importance of knowing one another personally in order to develop trust, knowledge of the competence of NPs is also vital in this respect [18,24].

In Norway, the implementation of the role in the health service is at an early stage. The NPs in this study believed that the lack of trust might arise from the fact that their role and education are new in Norway, and yet not very well known. Also, the absence of a clear role description impeded collaboration and could lead to confusion and resistance from GPs. Research from other countries that are more advanced than Norway in this respect has shown that implementing a new role that entails a new competence level for RNs has proved challenging in many places [9]. GPs scepticism is one of the barriers described. International research indicates that much of this opposition is apparently embedded in uncertainty about what the NP role entails, what competence they have and the GPs desire to protect their own profession [9,15]. When one party can rely on the other’s clinical competence, mutual trust and respect will develop and it will be less necessary to check each other’s contribution [25]. This may be one of the reasons why the informants in our study believed that collaboration between doctors and nurses in hospitals is better than in the primary healthcare service. In hospitals, nursing roles, including various specialized nursing roles, are well defined and established. Hospital nurses and doctors also often work more closely and know each other better, which naturally lead to greater trust from doctors in the competence of nurses.

Holm Hansen et al. [26] emphasized in their study of the introduction of NPs in out-of-Hours Medical Service that NPs must demonstrate their competence in actual patient situations to gain the trust of doctors. In step with the development of the profession in Norway, knowledge of their competence and skills, as well as how they can collaborate with GPs in patient treatment, will increase over time. With greater familiarity with the role, trust can be established that in turn will promote the development of forms of collaboration. Making the NPs competence more visible and trustworthy in the primary healthcare service might also encourage GPs to seek out NPs expertise by requesting patient information. However, this necessitates the creation of meeting points where such collaborative relationships can flourish.

The resent standardization of education with the Regulation on the National Guidelines for Master′s Education in Advanced Clinical General Nursing [27] will give the role more formal status and will probably be important for society’s trust. The introduction of specialist qualifications for NPs in 2022 will also promote this [28]. In addition to standardizing education, the importance of regulation and formalization of the role in the implementation process are highlighted in international literature [29]. In Norway, there is no further regulation of the role, consequently, it falls upon each employer to define the role and responsibilities of the NP. This makes it difficult to understand their scope of practice, function and role.

To catalyse the process of role development in Norway, a more uniform description is needed of what NPs can contribute to the health service, how their competence can be integrated in collaboration with existing professions, and how the role should be formalized. One way to regulate the role would be to specify the rights NPs should have in terms of independently contributing to the patient follow-up of certain patient groups. For instance, the informants in our study experienced that the lack of referral and prescribing rights meant that it was not possible to exploit the full benefits of their competence, further limiting their function. Clarification of their independent function must also entail shifting responsibility. Such a development would be in line with how NPs are employed internationally [9]. Some may argue that increased rights for NPs regarding referral and prescribing rights could strain the healthcare system by leading to more referrals and/or overmedication in certain patient groups due to NP involvement. However, international research shows that this does not pose a significant risk, as NPs tend to be cautious. At the same time, increased rights for NPs could improve access to healthcare services for vulnerable patient groups, which should be a compelling argument for exploring new ways of organizing municipal healthcare services. NPs have independent or limited prescriptive authority in several countries. Legislative changes have been required in these countries [30].

## Task allocation and new forms of collaboration

In the view of the informants, a lack of clarity in the demarcation between the spheres of GPs and NPs and their respective tasks impacted on collaboration. As can be expected, challenging professional boundaries triggers tension. The NPs in our study described that GPs didn’t want them to carry out or take over medical tasks. According to international research, doctors have traditionally been least willing to entrust tasks such as prescribing drugs, ordering blood tests and performing clinical examinations to NPs. These are regarded as being among the key tasks of doctors [31]. Opposition to NPs is strong when the reallocation of tasks means surrendering tasks previously ‘owned’ by doctors and which are part of the medical domain and responsibility [26,32].

When collaborating with healthcare personnel, the GP has the medical responsibility for the patient and makes final decisions regarding medical care and treatment according to the Health Personnel Act [33]. However, the Health Personnel Act does not clearly define what professions should perform what tasks but stipulates that healthcare personnel can assign certain tasks to other personnel if justified by the nature of the task, the qualifications of the assigned personnel and the follow-up provided [33]. Thus, there are no legal impediments to stop other personnel making a diagnosis, making clinical decisions, and performing tasks that are assigned by the doctor if they have the required qualifications and the task can be performed in a safe manner [34]. If this restriction on who is permitted to perform different tasks is extended in the tariff system, the tasks that NPs or other healthcare personnel can perform independently would be mainly based on an assessment of justifiable risk. More recent assessments show that greater use of interdisciplinary collaboration is vital in safeguarding increasingly complex patient needs, while the sustainability of the health service is conditional on the correct use of professional resources in the future [34,35]. Therefore, a reorganization of services that takes into account the need for collaboration between service providers is a measure that can also help to formalize the collaboration between NPs and GPs. Currently, for example, different models are being developed in Norway for NP teams, and both the organization and localization of these are probably important for the realization of established forms of collaboration. An Expert Committee has been convened by the Norwegian government to offer specific recommendations on the organization and financing aimed at enhancing the regular GP scheme. The Expert Committee’s recommendations for a sustainable health service in the future emphasize organizational and financial adjustments for interdisciplinary collaboration between GPs and other groups of healthcare personnel. These include both the incorporation of several healthcare personnel groups in the GP practice and the reallocation of tasks among the professions. Profession-neutral tariffs, legislative changes and the preparation of national guidelines for task reallocation in the GP service are among the proposed measures [34]. This will provide scope for NPs to establish constructive forms of collaboration with GPs to improve access to health services and promote an appropriate allocation of resources. There has been increased focus on the needs of vulnerable patients who have little contact with the health service with the aim of identifying and safeguarding them [34]. Homecare has many service users in this category. They have either physical problems preventing them from visiting their GP or mental challenges entailing that they see no need to visit their GP. NPs are mentioned in particular as a group that could give this group access to health services [34]. Homecare services primarily cater to frail geriatric patients who require a thorough assessment from an interdisciplinary team. However, these patients often struggle to physically visit their GPs office, weakening the doctor-patient bond. The regular GP-system is not adequate for patients who cannot actively seek their GP [36]. NPs, however, are uniquely positioned to fill this gap. With their specialized medical knowledge, NPs provide valuable insight into the patient’s overall health situation, including their living conditions, family situation, and other influencing factors. Furthermore, NPs ability to follow patients over time at home and collaborate with family members and other healthcare professionals offers a broader understanding of the patient’s health condition [24]. This is an important contribution currently missing in the healthcare system.

## Methodological considerations

The study’s five informants were all pioneer NPs in homecare in Norway. They came from four different municipalities and collaborated with various GPs, thus having broad and deep insight into the theme of the study. The interviewer is a NP with experience from the primary health service, and this background may have helped to elicit rich, detailed descriptions in the interviews that have shed light on the research question and raised interesting perspectives [12,37]. The researcher’s background as an NP likely provided valuable firsthand insights into NPs collaborative experiences with GPs, enriching the depth of data collection and analysis. However, it’s crucial to recognize the potential for bias stemming from the researcher’s professional identity. This necessitated ongoing reflexivity and consideration in terms of how being aware of own feelings and hypotheses, having an open mind during the interviews and analyzing process to ensure the study’s credibility and reliability. Accessing relevant participants was challenging due to the scarcity of NPs working in home care at that time in Norway, impacting the study’s sample size. Self-selection provide informants that are motivated to share their experiences, but this sampling method may also contribute to a non-representable sample [38]. While the sample size may seem small, qualitative research prioritizes depth over breadth, allowing for rich insights into participants experiences. Despite the modest sample size, rigorous analysis techniques aimed to ensure thorough exploration of themes and contribute meaningfully to understanding dynamics of NPs collaborative experiences with GPs [39]. A larger sample would have contributed further nuances and perspectives. The sample consists of NPs only from urban municipalities. There were only a few, and all of them urban, municipalities in Norway having NPs in defined positions. There is a pressing need for additional research focusing on rural municipalities that have recently integrated NPs. A weakness of the study is that it is limited to the perspectives of NPs on the collaboration, so that assertions about the GPs role must be interpreted with caution. Future studies should also explore GPs experiences of collaboration with NPs.

Although the interviewer has described and reflected on their prior understanding throughout the study, we cannot discount the risk of this affecting the interviews and the results of the study. The co-authors have clinical experience from different parts of the health services, and group discussions linked to the analysis and drafting of the material have helped to elicit different perspectives, strengthening the credibility of the study [12,37]. The results from this study have the potential to inform policy, shape practice guidelines, guide professional development, and stimulate research aimed at improving NP-GP collaboration, thus leading to enhanced patient care in community settings. The results can be transferred to similar healthcare contexts where NPs and GPs collaborate, such as other community care settings or primary care practices. Professionals working in these settings can relate to the challenges identified, such as organizational barriers and systemic factors affecting collaboration. Understanding the importance of trust and communication in collaboration can guide educational programs and training efforts. By developing trust, clarifying roles, and addressing systemic barriers, healthcare teams can work more effectively together to meet the various needs of patients in community care settings. Addressing legal frameworks and clarifying NP roles and responsibilities can enhance collaboration and optimize the use of NP expertise.

## Conclusion

Interpersonal dynamics, organizational structures and systemic frameworks collectively influence the trust and collaboration dynamics between GPs and NPs.

When establishing the role of the NP, trust in their competence is not fully established and thus personal trust is vital. When the NP role becomes more established and better known, and their rights and areas of responsibility clarified, generalized trust in the role will develop and collaboration will be less based on knowing each NP personally. Organizational structures such as a lack of meeting places, geographic and organizational divides, and limited communication all meant that it was difficult for GPs to get to know NPs. Consequently, the development of a professional relationship and trust took time which impeded collaboration. Moreover, systemic frameworks such as unclear demarcation between professional boundaries and unclear task allocation impacted on collaboration. Changes in legislation, the tariff system and guidelines on task allocation are measures that can change this. Collaboration is an important priority in national guidelines, but the study shows that some aspects must be changed or improved for such policies to produce results in practice.

Future research must investigate GPs experiences of collaboration with NPs. Role development in Norway proceeds apace, and research into NPs experience of GPs and how this has developed over time is important in identifying what promotes and inhibits collaboration. If new forms of task allocation or broader rights promote a more independent role and function for NPs in their collaboration with GPs, it is vital to follow this up through studies that will ensure quality and patient safety.

## Data Availability

The data that support the findings of this study are available on reasonable request from the corresponding author. The data are not public available due to privacy and ethical restrictions.
